# CO_2_ recycling by phospho*enol*pyruvate carboxylase enables cassava leaf metabolism to tolerate low water availability

**DOI:** 10.3389/fpls.2023.1159247

**Published:** 2023-05-09

**Authors:** Nattharat Punyasu, Saowalak Kalapanulak, Treenut Saithong

**Affiliations:** ^1^ Bioinformatics and Systems Biology Program, School of Bioresources and Technology, and School of Information Technology, King Mongkut’s University of Technology Thonburi (Bang Khun Thian), Bangkok, Thailand; ^2^ School of Bioresources and Technology, King Mongkut’s University of Technology Thonburi (Bang Khun Thian), Bangkok, Thailand; ^3^ Systems Biology and Bioinformatics Research Group, Pilot Plant Development and Training Institute, King Mongkut’s University of Technology Thonburi (Bang Khun Thian), Bangkok, Thailand

**Keywords:** carbon metabolism, genome-scale metabolic modeling, cassava leaf, cassava (*Manhiot esculenta*), drought response, phospho*enol*pyruvate carboxylase, CO2 recycling, PEPC

## Abstract

Cassava is a staple crop that acclimatizes well to dry weather and limited water availability. The drought response mechanism of quick stomatal closure observed in cassava has no explicit link to the metabolism connecting its physiological response and yield. Here, a genome-scale metabolic model of cassava photosynthetic leaves (leaf-MeCBM) was constructed to study on the metabolic response to drought and stomatal closure. As demonstrated by leaf-MeCBM, leaf metabolism reinforced the physiological response by increasing the internal CO_2_ and then maintaining the normal operation of photosynthetic carbon fixation. We found that phospho*enol*pyruvate carboxylase (PEPC) played a crucial role in the accumulation of the internal CO_2_ pool when the CO_2_ uptake rate was limited during stomatal closure. Based on the model simulation, PEPC mechanistically enhanced drought tolerance in cassava by providing sufficient CO_2_ for carbon fixation by RuBisCO, resulting in high production of sucrose in cassava leaves. The metabolic reprogramming decreased leaf biomass production, which may lead to maintaining intracellular water balance by reducing the overall leaf area. This study indicates the association of metabolic and physiological responses to enhance tolerance, growth, and production of cassava in drought conditions.

## 1 Introduction

Drought is an environmental stress crucially affecting the productivity of all major staple crops. The impact is more pronounced under exacerbating climate changes during the past decades. The circumstance necessitates new strategic development of drought-tolerant crops, for which an understanding of plant responses to stress at the metabolic level is essential to address the challenges of limited water availability for crop production and food insecurity (Kapoor et al., 2020).

Cassava (*Manihot esculenta* Crantz) has been recognized as an ideal crop to combat food insufficiency in the future, especially under the threat of climate change. Cassava starchy roots provide competitively high calories per cultivation area over other staple crops. Cassava roots contain up to 70-90 percent of starch on a dry matter basis. The main cultivation areas of cassava are in tropical and subtropical regions, though it can be grown in broad climatic conditions. Cassava is one of only a few crop plants that tolerate drought well. It can survive and produce reasonable yield even in marginal environments where other crops fail (El-Sharkawy, 1993; Howeler et al., 2013). Despite this, cassava production losses from unfavorable water conditions have been increasingly reported in the recent years (Vandegeer et al., 2013; Daryanto et al., 2016). There is a concerted effort to unravel the metabolic adaptation and physiological responses of cassava to water-deficit stress. Enhancing the current water use efficiency of cassava is a promising strategy to cope with the imminent drought and erratic rainfall due to climate change.

Physiological responses of cassava to drought stress have been studied for over three decades. When exposed to drought, cassava rapidly closes leaf stomata to reduce transpiration (El-Sharkaway and Cock, 1984)and also maintains intracellular water balance by reducing the overall leaf area under prolonged drought (El-Sharkawy and Cock, 1987b; El-Sharkawy, 2007). Photosynthesis and carbon fixation of cassava has been discussed in the possibility of containing the superior characteristics of C_3_-C_4_ an intermediate plant to drought response (Cock et al., 1987; El-Sharkawy and Cock, 1987a). The most recent studies of cassava leaf anatomy and photosynthetic physiology showed that cassava has more closed characteristics to a C_3_ plant (Edwards et al., 1990; Angelov et al., 1993; Arrivault et al., 2019), with an exceptional stomata sensitivity to air humidity that makes it tolerant to stressful environments (El-Sharkawy, 1993). In addition, cassava leaves can maintain reasonable photosynthetic rates and capacity even with the stomata closed in response to drought. While the metabolic response to drought has been less studied, it was shown that cassava metabolism might reinforce the physiological response through photosynthesis, photorespiration, and cellular CO_2_ re-assimilation (Alves and Setter, 2004). Particularly, the high activity of phospho*enol*pyruvate carboxylase enzyme (PEPC; a key enzyme in the C_4_ photosynthetic pathway) in cassava suggests its potential role in enhancing respiratory CO_2_ capture when intracellular gas exchange was limited by stomatal closure during water stress (Lopez et al., 1993; El-Sharkawy, 2007; El-Sharkawy et al., 2008). It was proposed to be involved in elevating CO_2_ availability around RuBisCO enzyme and hence lower oxygenase reaction (photorespiration) under stress conditions (Leegood, 2002). The highly correlated relationship among PEPC activity, photosynthetic performance, and cassava root yield under stress conditions marked the relevance of the enzyme in response to drought, supposedly *via* metabolic re-adjustment in leaves (El-Sharkawy et al., 2008). Transgenic rice with PEPC overexpression showed high tolerance to drought stress, increased net photosynthesis, altered sugar metabolism, and comparable yield to wild-type plants (Zhang et al., 2017). The PEPC enzyme is, therefore, potentially one key factor enabling cassava to maintain photosynthetic capacity and subsequent metabolic adaptation to retain yield under drought conditions (El-Sharkawy, 2007). Though contributions of the enzyme to cassava metabolism and also metabolic responses to drought stress have been postulated for years, in-depth studies on the mechanism remain absent. The role of the PEPC enzyme in CO_2_ re-assimilation is still ambiguous due to the incomplete Kranz anatomy of cassava leaves (Angelov et al., 1993). Furthermore, only a little is known about the synchronous action of the metabolic adaptation and physiological response supporting drought tolerance of cassava.

Elucidation of intracellular plant metabolism is a challenging research topic, and always requires intricate experiments with advanced instruments. Mathematical modeling has thus been introduced as an effective tool to assist in the laborious investigation of various aspects of the metabolic complexity (Hädicke et al., 2011; Collakova et al., 2012). Constraint-based metabolic modeling (CBM) *via* flux balance analysis (FBA) allows the prediction of steady-state reaction fluxes in metabolism by applying mass balance constraints. FBA calculates optimal fluxes for biomass production, predicting the growth and production rates of biotechnologically important compounds under conditions of interest (Orth et al., 2010). This approach provides a new outlook on controlling the pleiotropic functionality of a system and enables hypothesis testing about mechanisms underlying the function of cells (Oberhardt et al., 2009). During the last ten years, constraint-based modeling was used to investigate the complexity of primary carbon metabolism in broad plant species, including *Arabidopsis* (Beckers et al., 2016), barley seed (Grafahrend-Belau et al., 2009), maize (Saha et al., 2011), rice (Lakshmanan et al., 2013), and soybean (Moreira et al., 2019). The approach was also applied to gain insights into metabolic responses in various conditions, such as *in silico* flux analysis of rice leaf cells which suggested an increase in PEPC activity, photorespiration and nitrogen assimilation under severe drought conditions (Lakshmanan et al., 2013). In cassava, CBM was applied to examine carbon assimilation toward root biomass biosynthesis to understand the difference in storage root production in high- and low-yielding cassava varieties (Chiewchankaset et al., 2019). Though critical to plant growth and root development, there are just a few studies on metabolism in photosynthetic tissues of cassava, including the reconstruction of a compartmentalized metabolic network of primary carbon assimilation (Punyasu et al., 2019; Sutthacharoenthad et al., 2022).

Here, computational analysis was performed to study the response of cassava leaf metabolism under drought conditions. The study also investigated the involvement of PEPC in supporting the robustness of leaf carbon metabolism against drought stress. In this work, central carbon metabolism related to cassava leaf growth was reconstructed mainly based on a genome-based metabolic network of cassava (Punyasu et al., 2019), available metabolic models of plants, and the current physiological knowledge on cassava photosynthesis and carbon assimilation. The constructed model, namely leaf-MeCBM, was modeled to imitate the photoautotrophic characteristics of cassava leaves, whose growth primarily depended on atmospheric CO_2_ fixation during photosynthesis. Based on leaf anatomy of cassava that follows typical C_3_ leaf characteristics, the leaf-MeCBM represents primary metabolism in a mesophyll cell including four sub-cellular compartments, cytosol, chloroplast, mitochondria, and peroxisome. It consists of 424 reactions (564 cassava genes) related to 10 pathways, including photosynthetic light-dependent reactions, Calvin-Benson cycle, photorespiration, respiration, pentose phosphate, nitrogen assimilation, starch and sucrose metabolism, amino acid metabolism, primary cell wall metabolism, and fatty acid synthesis. The leaf-MeCBM was first verified by simulations to represent cassava leaf growth under normal conditions. The leaf-MeCBM was subsequently used to investigate metabolic responses to drought by restraining the rate of CO_2_ exchange to the environment (i.e., to represent the stomatal response to a dry environment). The resulting high fluxes through PEPC and RuBisCO reactions set the initial hypothesis of the study to investigate the involvement of PEPC in metabolic adaptation under drought conditions. Through *in silico* perturbation of PEPC activity, flux distribution analysis was performed to study the association of enzymatic reactions in metabolism and the physiological response to stress. The model prediction was finally validated based on the related expression of enzymatic genes and the collective evidence in cassava literature. Comprehension gained in this study would bridge the gap on how metabolic and physiological responses act in concert to boost tolerance to drought conditions.

## 2 Materials and methods

### 2.1 Model construction and verification

The leaf-MeCBM model was constructed primarily based on the reported metabolic network of cassava photosynthetic tissues (ph-MeReCon), which contains nine biochemical pathways (i.e., photosynthetic light reaction, Calvin cycle, respiration (glycolysis, TCA, oxidative phosphorylation, and fermentation), pentose phosphate pathway (PPP), photorespiration, starch and sucrose metabolism, and anabolic pathways of amino acids, fatty acids, fibers, and nucleic acids)(Punyasu et al., 2019). Additional metabolic pathways, PEPC-related reactions and drought-responsive pathways, were first taken from the published metabolic models of *Arabidopsis* (Beckers et al., 2016), and rice (Lakshmanan et al., 2013), and verified by the existence of their enzymatic proteins in the cassava genome (BLASTP, using the following criteria: (1) E-value ≤ 1x10^-10^, (2) identity percentage ≥ 60, and (3) coverage percentage ≥ 80). Reactions related to drought-responsive pathways (e.g., energy, redox and nitrogen metabolism), from the published metabolic models of *Arabidopsis* (Beckers et al., 2016) and rice (Lakshmanan et al., 2013), were also included in leaf-MeCBM. The entire biochemical reactions were also verified based on the expression of their responsible genes in cassava fully expanded leaves. The model connectivity was analyzed using GapFind/GapFill algorithms. All gaps were manually curated according to the published information and the available biochemical data in KEGG (Kanehisa et al., 2008) (https://www.genome.jp/kegg/) and Plant Metabolic Network (Schläpfer et al., 2017) (PMN, https://plantcyc.org), databases.

To represent biomass production in photosynthetic tissues, we ran the model by assuming that inorganic compounds such as CO_2_, photons, oxygen, water, phosphate, nitrogen, and sulfate were freely exchanged between the system and the external environment. Biomass synthesis was calculated based on biomass composition in cassava leaves, including carbohydrate, protein, fiber, and lipid (Bradbury and Holloway, 1988). The model was optimized by maximizing leaf biomass production using a similar method proposed by Moreira et al. (2019). Flux balance analysis (FBA) was carried out using COBRA toolbox (Heirendt et al., 2019) running on the MATLAB environment. The objective function was maximizing the growth rate of cassava leaves subjected to constraints (Equation 1): (i) steady state, (ii) uptake of light energy (photon) based on the maximum light use efficiency [LUE (Santanoo et al., 2019)], and (iii) the energy demand for cellular maintenance. The simulated carbon fluxes were predicted when the simulation satisfied the physiological constrained criteria i.e., a specific growth rate (rate of biomass production) of cassava leaves.


(Equation 1) $$\begin{array}{c} \max\;Z_{biomass}=\;\sum_{j}\;C_{j}V_{j}\\ subjected\;to:\;\;\;\sum \underset{j}{i}S_{ij}.v_{j}=0\\ LB\;\leq v_{j}\;\leq UB \end{array}$$

Where Z corresponds to the objective function of the system, where the relative weights are determined by the objective coefficient c_j_. $S_{ij}$ refers to the stoichiometric coefficient of metabolite *i* participating in reaction *j*. $v_{i}\;$ denotes the flux of reaction *j*. *LB* and *UB* are lower and upper boundaries of the flux of reaction *j*, respectively.

The leaf-MeCBM was used to simulate carbon metabolism inside cassava leaves according to data of cv. Kasetsart 50 (KU50) grown in greenhouse conditions (in-house unpublished data). The uptake rate of carbon (CO_2_) was constrained to the experimentally measured values. For the scenario-based simulation of drought conditions, flux through the PEPC reaction was increased by ten times the normal condition. The resulting simulations were expressed both in terms of flux quantity and relative flux ratios.

Here, the resulting leaf-MeCBM model was verified to represent the photoautotrophic metabolic process in typical leaves of C_3_ plants and be able to simulate realistic responses to stress. The former was done by comparative analysis of the leaf-MeCBM model simulation with available literature data. Scenario-based simulation of photorespiration was performed. Here the ratio of carboxylation to oxygenation varied from V_c_ : V_o_= 1 (severe drought, more O_2_ fixation) to V_c_ : V_o_ = 5 (normal conditions) using the same set of model constraints. The patterns of flux fold changes in central metabolism and photorespiration-related pathways were examined for concordance with the predicted metabolic responses of plant leaves to different levels of photorespiration (Lakshmanan et al., 2013).

### 2.2 Flux-sum analysis

Flux-sum analysis was performed to evaluate the metabolite pool, which is indicative of the metabolite turnover rate (Chung and Lee, 2009) and metabolic perturbations. The flux-sum of metabolite *i* ( $x_{i}$ ) was calculated from one-half of the summative production and consumption fluxes of the individual metabolite under a steady-state condition (Equation 2).


(Equation 2) $$x_{i}=0.5\sum_{j}\;\left|S_{ij}v_{j}\right|$$

where $S_{ij}$ refers to the stoichiometric coefficient of metabolite *i* participating in reactions *j*. *v_j_* is the flux of reaction *j*.

### 2.3 Transcriptome data analysis

In this study, the gene expression data was taken from a publicly available leaf RNA-seq dataset of cassava cv. KU50 treated with 20% PEG 6000 solution for 3 and 24 hours (Ding et al., 2019) (NCBI SRA accession no. SRP162280). The taken sequencing reads were trimmed the adaptor sequences and removed low -quality and contaminated reads. The resulting high-quality reads were aligned with the cassava reference genome version 6.1 provided on the Phytozome database using the STAR aligner (Dobin et al., 2013). The reads were normalized using GeTMM (Smid et al., 2018) and then used to quantify the expression levels of the metabolic genes. Positive and negative log_2_FC indicated up- and down-regulated expression under drought stress relative to normal conditions, respectively.

## 3 Results

### 3.1 Modeling carbon assimilation in cassava leaves

A constraint-based metabolic model of cassava leaves, namely leaf-MeCBM, was constructed to represent basic carbon metabolism underlying the growth of cassava leaves under adequate water availability. The model described the primary carbon metabolism in photosynthetic tissues that convert carbon dioxide and light energy to cellular biomass (i.e., carbohydrates, proteins, fibers, and lipids) and photosynthates (i.e., sucrose). It was constructed based on a genome-based metabolic pathway of primary carbon metabolism in cassava leaves (ph-MeRecon (Punyasu et al., 2019), https://bml.kmutt.ac.th/ph-MeRecon/) and curated by information from published metabolic models of *Arabidopsis* leaves (Beckers et al., 2016) and rice leaves (Lakshmanan et al., 2013). All metabolic reactions and the associated enzyme genes were verified for existence in the cassava genome. Developed through an iterative process of pathway reconstruction (see “Methods”), the leaf-MeCBM model contains 523 metabolites and 424 reactions (564 genes) related to 10 pathways, including photosynthetic light-dependent reactions, Calvin-Benson cycle, photorespiration, respiration, pentose phosphate, nitrogen assimilation, starch and sucrose metabolism, amino acid metabolism, primary cell wall metabolism, and fatty acid synthesis (**Supplementary Figure 1**). These metabolic pathways were assumed to function across four subcellular compartments i.e., cytosol, chloroplast, mitochondria, and peroxisome (**Supplementary Table 1**), with 46 transport reactions interconnecting metabolic conversions across subcellular locations. The model is provided in **Supplementary Data 1**.

The leaf-MeCBM was modeled to imitate the photoautotrophic characteristics of cassava leaves, whose growth primarily depended on atmospheric CO_2_ fixation during photosynthesis (**Supplementary Figure 2**). The net photosynthetic CO_2_ uptake rate was derived from the measured photosynthetic rate (P_N_). The corresponding photon uptake rate was estimated based on the light use efficiency (LUE) of cassava under well-watered conditions (Sutthacharoenthad et al., 2022). The carboxylation to oxygenation ratio of RuBisCO was set to 3:1 (V_c_/V_o_=3) as observed in a typical ambient condition (Jordan and Ogren, 1984; Sharkey, 1988). Given condition-specific data, the leaf-MeCBM-simulated synthesis rate of leaf biomass fitted the measured leaf growth rate of cassava grown in a semi-closed greenhouse environment (in-house unpublished data). The simulated flux distribution agreed with the typical metabolic process of C_3_ plants and also of cassava (**Supplementary Table 2**). The model could replicate photosynthesis and carbon fixation, including the conversion of light energy to biochemical energy and CO_2_ conversion to carbohydrate molecules (Baslam et al., 2021). Cellular respiration, including glycolysis, tricarboxylic acid (TCA) cycle and mitochondrial electron transport chain, were functionally active to generate energy for cellular activities. The model also simulated the reasonable occurrence of photorespiration, the loss of carbon to the RuBisCO oxygenation pathway. Refixation of photorespiratory ammonia (NH_3_) through amino acid biosynthesis (GS/GOGAT cycle) was also found in the leaf-MeCBM (Bauwe et al., 2010). The interdependence of the photorespiration and nitrogen assimilation highlighted an interaction between carbon and nitrogen metabolism *via* glutamate utilization and ammonia formation during the operation (Bauwe et al., 2010). Lastly, the activity of PEPC, a key enzyme in C_4_ photosynthesis present in cassava, was also captured by the leaf-MeCBM. In summary, the leaf-MeCBM adequately represented the metabolic behavior in cassava leaves underlying their growth under normal conditions.

**Figure 1:**
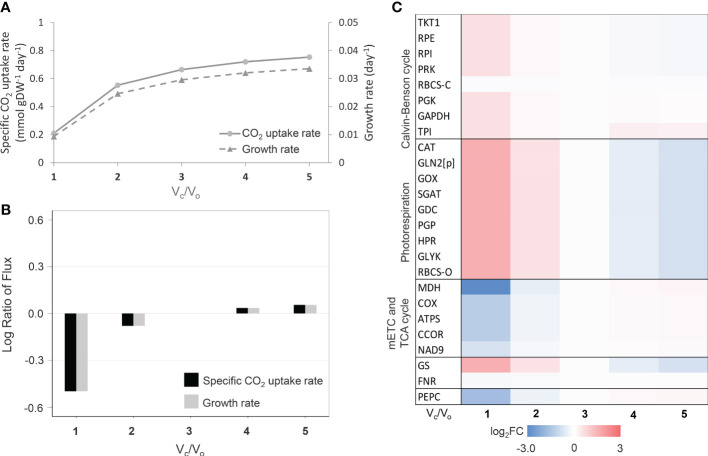
Simulation of leaf-MeCBM response at different photorespiration levels. **(A)** Simulated fluxes of CO_2_ uptake and cassava leaf growth rates. **(B)** Flux ratio of CO_2_ uptake and growth rates relative to V_c_/V_o_ =3 (representing normal conditions). **(C)** Changes in key metabolic fluxes through primary pathways related to photorespiration. Heatmap of log_2_ flux change relative to normal conditions (V_c_/V_o_ =3). The enzymes are abbreviated in blue and are listed in **Supplementary Table 3**.

Besides leaf growth characteristics, the leaf-MeCBM was employed to simulate the metabolic response to increased photorespiration, which happens in consistent with water-limiting environments. During CO_2_ fixation, photorespiration occurs when the RuBisCO enzyme selectively takes O_2_ (oxygenation; V_o_), instead of CO_2_ (carboxylation; V_c_). The process generally occurs in the leaf mesophyll at a high O_2_ to CO_2,_ typically during stomatal closure in response to stress. Here, we simulated the level of photorespiration by decreasing the carboxylation to oxygenation ratio of RuBisCO (V_c_/V_o_), from a low (V_c_/V_o_ = 5) to high (V_c_/V_o_= 1) degree of photorespiration; the ratio is approximately equal to 3 (Jordan and Ogren, 1984) in normal conditions. Model simulation showed the CO_2_ uptake rate declined with photorespiration (decreasing V_c_/V_o_ ratio, **Figure 1A**), which led to a substantial reduction in growth rate by up to 68.3% (V_c_/V_o_=1) relative to normal conditions (V_c_/V_o_=3) (**Figure 1B**). Changes in metabolic fluxes in response to the level of photorespiration were illustrated in **Figure 1C**. Fluxes of key metabolic pathways related to photorespiration were presented relative to those in normal conditions. At a high level of photorespiration (V_c_/V_o_<< 3), fluxes of the photorespiration-related reactions (e.g., RBCS-O, PGP, GOX, GGAT, and HPR) were more active, corresponding to the increased fluxes in the nitrogen assimilation pathway (i.e., GS-GOGAT cycle) and down-regulation of oxidative phosphorylation and TCA cycle. The highly active photorespiration pathway led to the higher ammonia produced and assimilated through GS/GOGAT cycle. The higher glycine decarboxylase (GDC) reaction flux, a part of photorespiration, supplied the reducing equivalent molecules (i.e., NADH) for the mitochondrial electron transport chain (i.e., COX, ATPS, and CCOR), leading to metabolic adjustment within the mitochondria to level down the activity of TCA cycle (i.e., MDH). The simulated flux patterns were consistent with studies on leaf metabolism in rice (Lakshmanan et al., 2013). In addition, the model showed that the reaction flux of PEPC decreased at higher photorespiration (**Figure 1C**). These predictions corresponded to the observed metabolic response to drought stress in C_3_ plants, as in this circumstance, leaf metabolism was altered by an increased O_2_/CO_2_ ratio as a consequence of stomatal closure. The leaf growth and metabolic responses to the degree of photorespiration captured in the leaf-MeCBM model ensured its representativeness of cassava leaf metabolism.

### 3.2 PEPC helped maintain carbon fixation and metabolic adaptation under drought conditions

**Figure 2:**
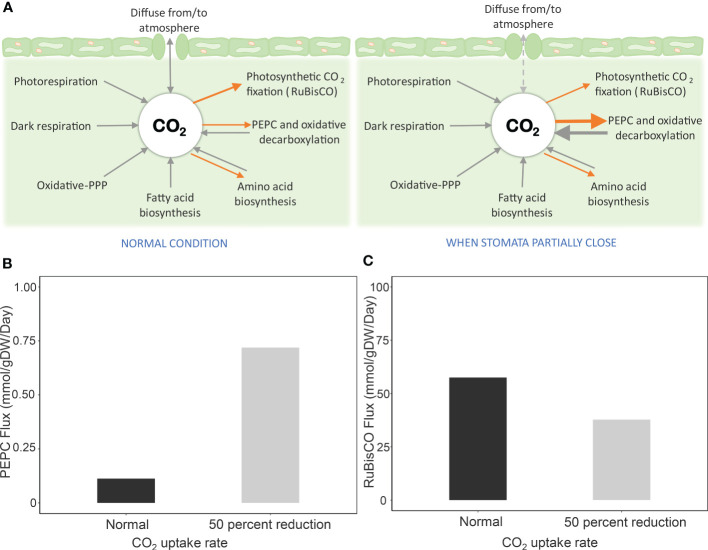
Simulation of PEPC reaction flux and carbon fixation by RuBisCO during stomatal closure. Drought stress was simulated by reducing the CO_2_ uptake rate by 50% due to partial stomatal closure. **(A)** Scheme of processes related to CO_2_ pool in leaves during stomatal opening and closing. Processes for generating CO_2_ are represented in black arrows, and CO_2_-consuming processes are indicated by orange arrows. When CO_2_ diffusion from the atmosphere is limited by stomatal closure, the highly active PEPC enzyme assimilates intracellular CO_2_ and later releases CO_2_ through oxidative decarboxylation for fixing by RuBisCO. Metabolic fluxes of **(B)** PEPC reaction and **(C)** carbon fixation by RuBisCO at a 50% CO_2_ uptake rate, relative to normal conditions, to simulate stomatal closure.

The contribution of PEPC to CO_2_ re-fixation was investigated by assuming that its reaction flux was more active and potentially played a role in providing CO_2_ to RuBisCO under limited CO_2_ diffusion conditions. **Figure 2** showed that the reaction flux of PEPC increased with reducing CO_2_ uptake rate (more severe stress). The flux was about six times higher at 50% reduction of CO_2_ uptake (**Figure 2B**), while the corresponding carbon fixation was retained at 66% compared to the normal condition (**Figure 2C**). Further investigation at a low V_c_/V_o_ ratio and one-half of CO_2_ uptake showed increased PEPC flux – i.e., lower V_c_/V_o_ = higher PEPC flux (**Supplementary Figure 3**). The results were consistent with the study of Lopez et al., who reported an increase of PEPC activity in leaves of drought-exposed cassava, with ~ 58% RuBisCO activity in carbon fixation (Lopez et al., 1993). Simulations were conducted to unravel the mechanistic role of PEPC in supporting cassava leaf metabolism to cope with water-limiting conditions.

#### 3.2.1 PEPC enzyme enhanced CO_2_ fixation via RuBisCO

**Figure 3:**
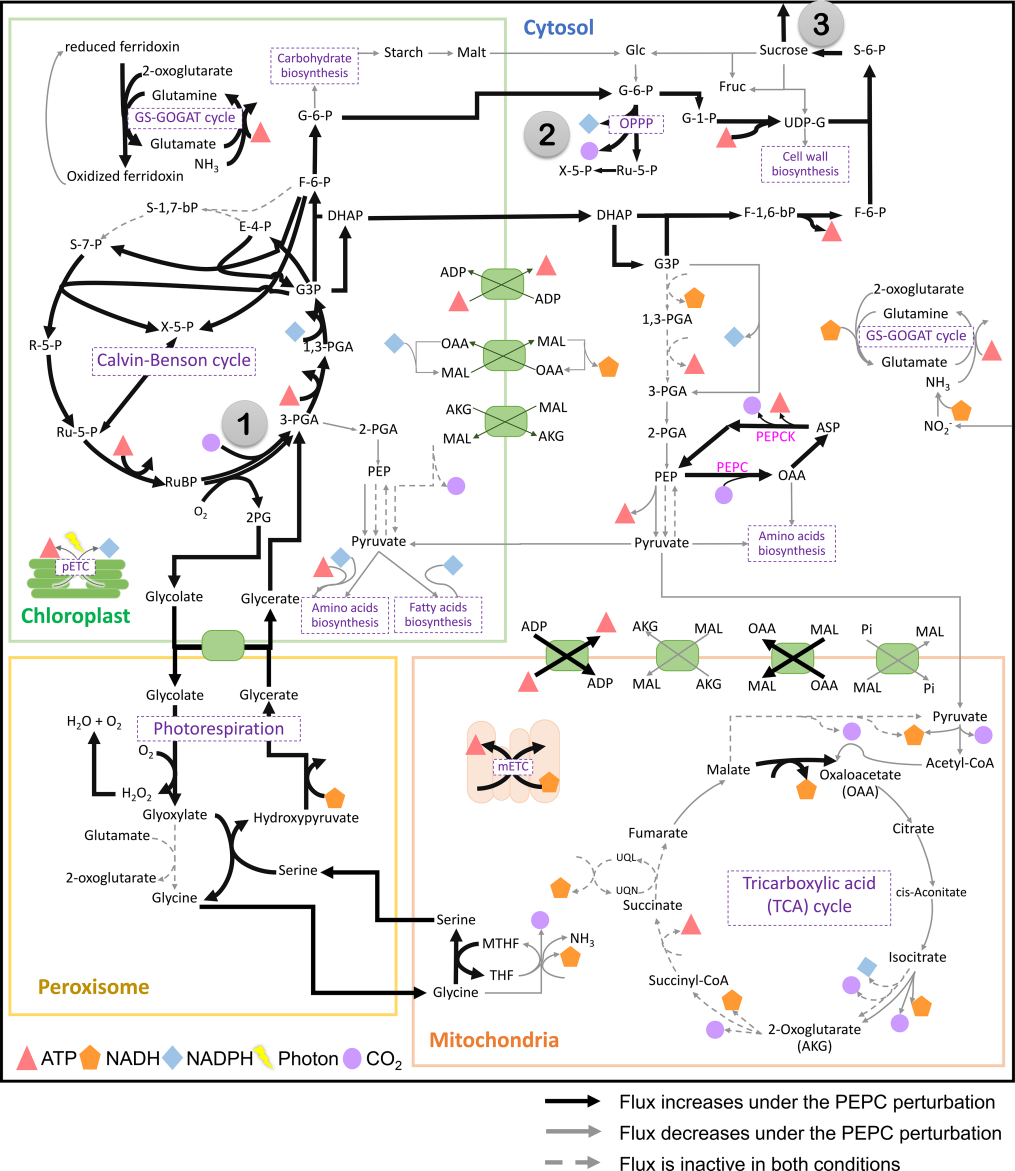
The simulated flux activity of the primary carbon metabolism in cassava leaves. The results are presented by contrasting the elevated PEPC scenario with the normal condition. The numbers in gray circles mark key points in metabolism: (1) RuBisCO reaction, (2) oxidative pentose phosphate pathway (OPPP), and (3) sucrose synthase and sucrose export reactions. Bold black and thin gray lines indicate the increased and decreased reaction fluxes under the PEPC perturbation, respectively, compared to normal conditions. The dashed gray line represents the inactive flux in both conditions. Symbols refer to energy metabolites (ATP, and NAD(P)H), photons, and CO_2_. Details of the compartments and reactions are provided in **Supplementary Figure 1**.

**Figure 4:**
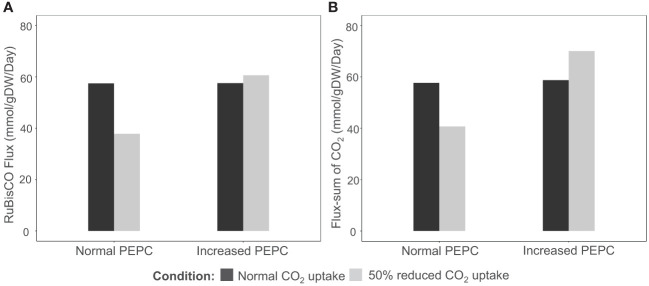
Simulation of **(A)** carbon fixation (RuBisCO) and **(B)** intracellular CO_2_ pool in response to PEPC perturbation (10 times increment) and a reduction in CO_2_ uptake rate (-50%). The black bars represent normal conditions (unconstrained CO_2_ uptake rate), and the grey bars indicate the scenarios of CO_2_ uptake perturbation.

PEPC was hypothesized to help maintain the carboxylation to oxidation ratio (V_c_/V_o_) of RuBisCO under drought stress by recycling the respiratory CO_2_ to the fixation reaction. The possible pathway was highlighted based on the observed upregulation of carbonic anhydrase protein (CA)(Chang et al., 2019), an enzyme involved in HCO_3_^-^ production for PEPC, corresponding to the increase in PEPC activity under drought conditions (Lopez et al., 1993; El-Sharkawy et al., 2008). In this study, we examined whether the proposed pathway feasibly occurs in the metabolism of cassava leaves during the stress response. *In silico* perturbation of PEPC activity was performed to simulate the metabolism under drought stress. By 10-times increase of PEPC activity, the phospho*enol*pyruvate carboxykinase (PEPCK) reaction increased its flux and the amount of intracellular CO_2_, suggesting higher activity of PEPC in the decarboxylation pathway (**Figure 3**). The accumulated CO_2_ induced metabolic adjustments, including higher carbon fixation by RuBisCO (**Figure 4A**). Flux-sum analysis of CO_2_ also indicated an increment in the CO_2_ turnover rate inside leaves (**Figure 4B**). These results highlighted the positive correlation of PEPC activity to CO_2_ re-assimilation by RuBisCO enzyme, even though the atmospheric CO_2_ uptake rate was limited. The simulated results demonstrated that PEPC acted in concert with RuBisCO to retain the carbon assimilation capacity under limited uptake of atmospheric CO_2_.

#### 3.2.2 Upregulating PEPC increased intracellular CO_2_ concentration and induced oxidative pentose phosphate pathway

**Figure 5:**
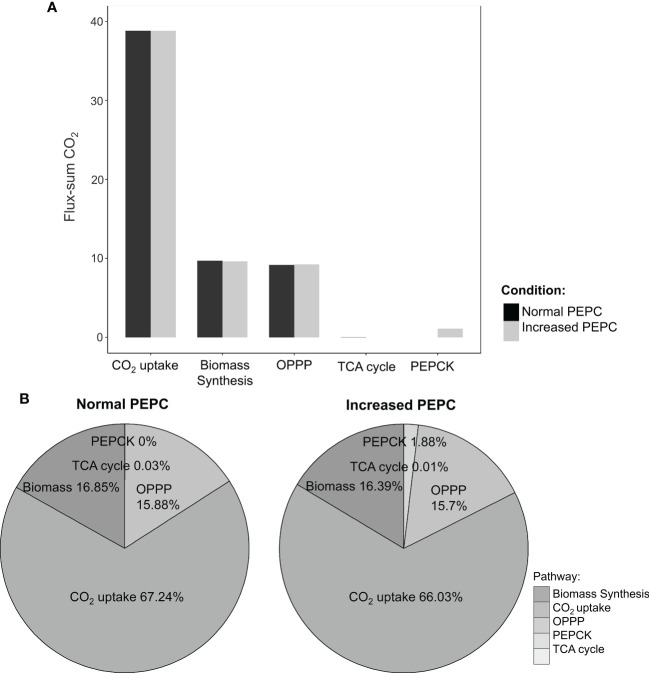
Simulation of intracellular CO_2_ production under normal and elevated PEPC conditions. **(A)** The bar graph shows the ratio of CO_2_-generating fluxes through key pathways between the elevated PEPC and the normal conditions. **(B)** Contribution of the primary pathways to CO_2_ pool under normal and increased PEPC conditions.

Further, sources of the increasing CO_2_ pool in response to PEPC perturbation were investigated. We found that the increased CO_2_ pool was contributed mainly by the phospho*enol*pyruvate carboxykinase (PEPCK) reaction and oxidative pentose phosphate pathway (OPPP) (**Figure 5A**). The PEPCK reaction reversely converted oxaloacetate (OAA) to phospho*enol*pyruvate (PEP) and released CO_2_. Our simulation found that the elevated PEPC activity increased CO_2_ generation through the PEPCK, accounting for an approximate 1.88% increase in the CO_2_ pool relative to normal conditions (**Figure 5B**). Another source of the CO_2_ pool was the higher cytosolic oxidative pentose phosphate pathway (OPPP) activity. The flux through OPPP was increased from 9.17 to 9.23 mmol/gDW/day in response to the increased PEPC activity (**Figure 3**). The more active cytosolic-OPPP enhanced reducing equivalents in the form of NADPH. The induced OPPP activity was postulated to achieve the biochemical supply for metabolization of the accumulated intracellular CO_2_. That might be a reaction to compensate for the lower NADPH generation from cytosolic glycolysis (glyceraldehyde-3-phosphate dehydrogenase (NADP^+^)) and the lower catabolic pathway activity as indicated by the reduced CO_2_ flux from biomass biosynthesis and the tricarboxylic acid (TCA) cycle under PEPC perturbation (**Figure 3**). Overall, the model simulation showed that changes in intracellular CO_2_ altered the energy and redox balance, which possibly affected subsequent metabolic adjustments to maintain the energy and redox status of the system.

#### 3.2.3 Increased PEPC activity elevated energy costs in chloroplast and cytosol

**Figure 6:**
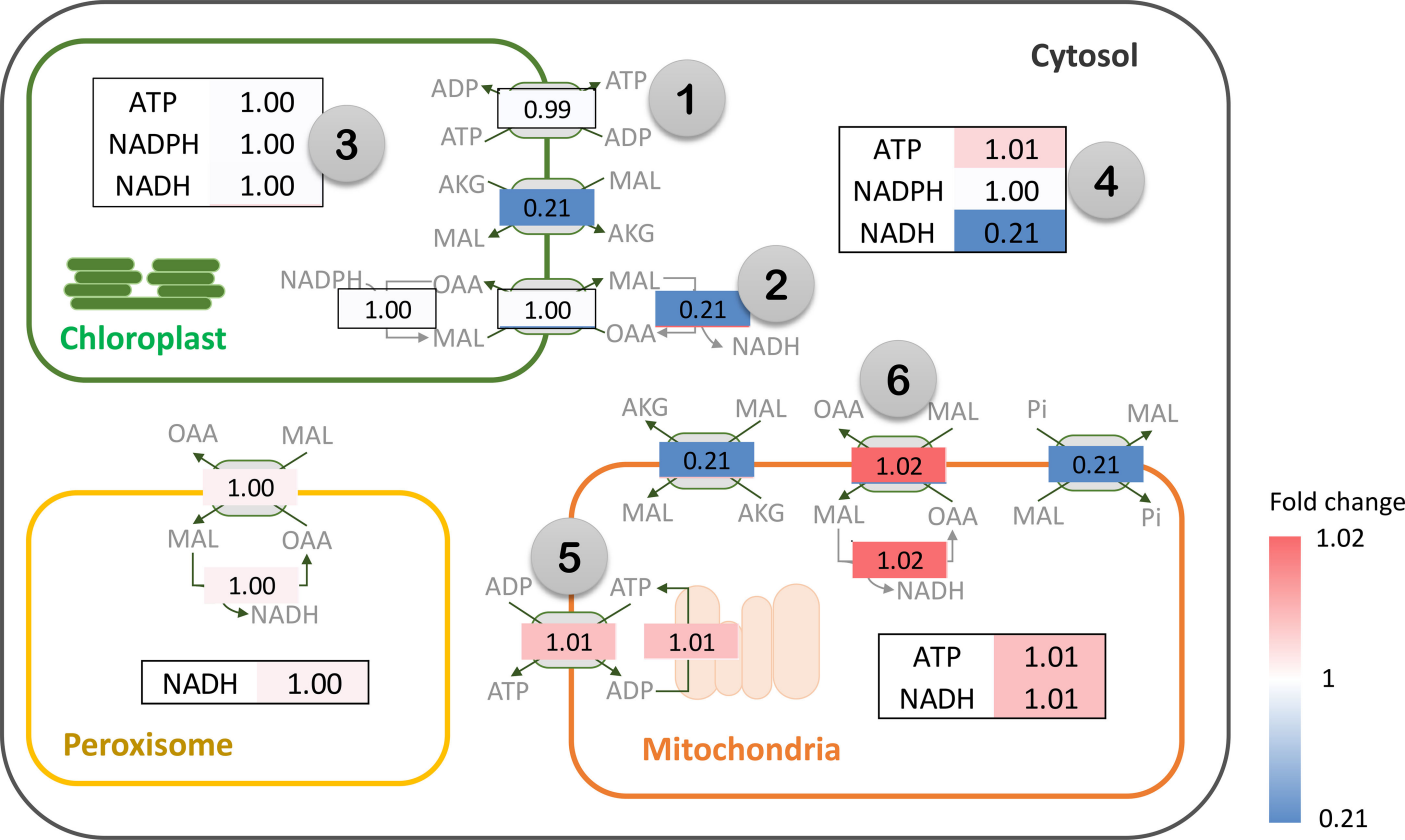
Simulation of energy (ATP) and redox (NAD(P)H) turnover rates (flux-sum) under the elevated PEPC activity in cassava leaves. The numbers represent the fold change of ATP and NAD(P)H turnover rates in the *in silico* PEPC upregulation scenario relative to normal conditions (Flux_PEPC_/Flux_normal_). Flux fold change of energy and redox translocators are shown in gray boxes. The color bar indicates the magnitude of flux fold change, ranging from blue (low) to red (high). Note that NAD(P)H is transferred across intracellular membranes using the malate-oxaloacetate (MAL/OAA) shuttle system.

Metabolic energy and redox conditions changed globally in the simulated drought-response metabolism. An increase in PEPC activity affected ATP metabolism and the redox (NAD(P)H) balance, which crucially influenced the metabolic status by modulating the interrelationship between catabolic and anabolic processes in leaf metabolism. Our simulation showed that metabolic processes in chloroplast were more energy intensive under increasing PEPC activity, attenuating the energy allocated to the cytosol as a result (**Figure 6**, Number 1: ATP/ADP and malate/OAA shuttles on chloroplast membrane). ATP and NADPH turnover rates in the chloroplast were found to be slightly higher under perturbed conditions, yet they were enough to reduce the energy supply (i.e., NADH) to the cytosolic metabolic pathway by approximately 5 times (**Figure 6**, Number 2: NADH translocation between chloroplast and cytosol and its turnover rate). Simulated flux distribution suggested that carbon fixation through the Calvin-Benson cycle required greater amounts of ATP and NADPH under the perturbed condition, and so did the downstream pathways, i.e., nitrogen assimilation (through GS/GOGAT cycle), amino and fatty acids biosynthesis, (**Figure 6**, Number 3, flux ratio ≥ 1). Besides the chloroplast, cytosolic metabolism also proved a high energy-demanding process as expressed by the greater net ATP turnover rate in this condition (**Figure 6**, Number 4). Simulations showed that cytosolic metabolism responded to the decreasing energy supply from chloroplasts by re-adjusting the high-energy metabolite allocation across metabolic processes, for example, reconciling the energy supply to nitrogen assimilation to maintain biochemical activity in peroxisomes (**Figure 3**). In this condition, the energy requirement of cytosolic metabolism was subsidized by redox pathways in the mitochondria (**Figure 6**, Number 5: ATP translocation through ATP/ADP shuttle). The higher cytosolic requirement of ATP induced metabolic reprogramming of redox pathways in mitochondria, seemingly fueled by the allocated malate from the cytosol (**Figure 6**: Number 6: malate allocation through mitochondrial malate/OAA shuttle). The predicted flux distribution demonstrated the higher activity of TCA and mitochondrial electron transport chain reactions (mETC) in converting NADH to ATP for alleviating a lack of ATP in cytosolic metabolism under elevated PEPC activity (simulated closed-stomata conditions). These highlighted the interchange of energy-sourcing processes in cassava leaf metabolism, from photosynthesis in chloroplasts under normal conditions to mitochondrial redox pathways under drought conditions.

Overall, the rebalancing of metabolic energy costs was associated with carbon metabolism. In response to the increased PEPC activity, the metabolism was globally adjusted towards maintaining redox and energy homeostasis at a high CO_2_ level. These changes affected the catabolic and anabolic relationship in cellular metabolism. Since the elevated PEPC reaction activated CO_2_ reassimilation by RuBisCO and flooded carbon fluxes through nitrogen assimilation, amino acid biosynthesis, and sucrose metabolism, the imbalance in energy demand and supply led to metabolic reprogramming toward improving the efficiency of energy usage during the response to the perturbation. It implied that the change in carbon balance mediated by the elevated PEPC activity impacted the metabolic state, especially in energy and redox balance, which has consequences for plant growth and development under drought-stress conditions.

#### 3.2.4 Increased PEPC activity affected carbon-nitrogen assimilation in metabolism

**Figure 7:**
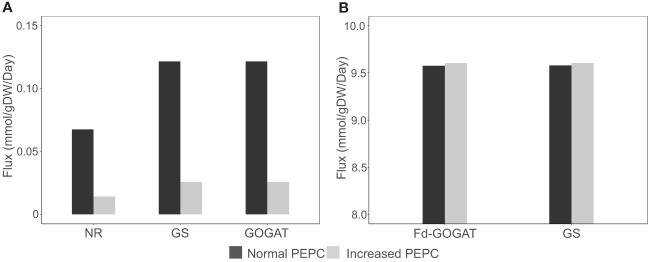
Simulation of metabolic fluxes in nitrogen assimilation pathway. **(A)** Simulated fluxes of nitrate reductase (NR), glutamine synthase (GS), and glutamate synthase (GOGAT) in the cytosol. **(B)** Fluxes of GS and ferredoxin-dependent glutamate synthase (Fd-GOGAT) in chloroplast under increased PEPC activity.

The redox status adapted for metabolic homeostasis altered the balance of carbon and nitrogen assimilation in the metabolism at high PEPC activity. The simulations showed a reduction in inorganic nitrogen assimilation *via* nitrate reductase (NR) (**Figure 7A**) and a subsequent decrease in the assimilation of ammonium (ion) into amino acids in the cytosol *via* glutamine synthetase (GS) and glutamate synthase (GOGAT) (**Figure 7A**), demonstrating energy optimization in cells during stomatal closure.

The change in carbon fluxes throughout metabolism also affected photorespiration and its related pathways. The elevated photorespiration at increased PEPC promoted C_1_ (tetrahydrofolate derivatives) metabolism through nitrogen-related pathways. We found that the higher fluxes through the photorespiration pathway in mitochondria required higher amounts of the tetrahydrofolate derivatives as cofactors (**Figure 3**). The metabolism then responded to the tetrahydrofolate requirement by inducing fluxes through the GS/Fd-GOGAT cycle (nitrogen metabolism) and serine metabolism in chloroplast (**Figure 7B**). The requirement of nitrogen skeleton, energy, and redox to operate the more active photorespiration may contribute to the metabolic adjustment toward energy and resource optimization.

#### 3.2.5 Increased PEPC activity affected carbon assimilation and photosynthate synthesis

**Figure 8:**
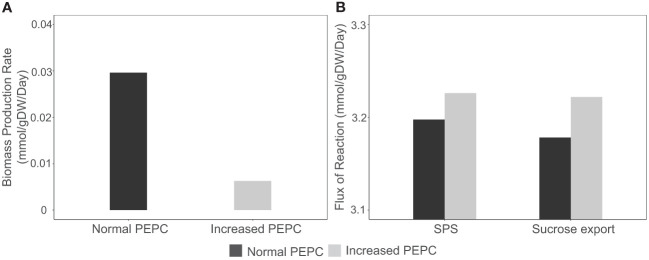
Simulation of biomass production rate and sucrose biosynthesis during response to in silico PEPC perturbation. **(A)** Simulated fluxes of cassava leaf biomass production under normal (black bar) and elevated PEPC conditions (gray bar). **(B)** Metabolic fluxes of sucrose biosynthesis *via* sucrose phosphate synthase (SPS) and sucrose export reaction during the increased PEPC activity.

The increasing CO_2_ pool and changes in energy and redox states induced metabolic reprogramming and resulted in metabolic tread-off between biosynthesis of cellular biomass and photosynthates (sucrose). Simulations showed the redirection of carbon fluxes towards sucrose biosynthesis rather than biomass production. At PEPC conditions, biomass biosynthesis pathways, namely amino acids, lipids, and carbohydrates, had relatively low fluxes compared to normal conditions, resulting in reduced biomass production rates of up to 79 percent (**Figure 8A**). On the other hand, carbon flux from the RuBisCO enzyme tended to be utilized for triose phosphate production, which was subsequently metabolized toward sucrose biosynthesis in the cytosol (**Figure 8B**) for utilization in metabolic processes in sink tissues. In sum, *in silico* upregulation of PEPC at simulated drought conditions showed high carbon partitioning in leaves for sucrose generation, which would be likely spent on sink metabolism (reserves), while limiting biomass production for leaf growth.

### 3.3 Meta-transcriptome supported the hypothetical role of PEPC to enhance metabolic response to drought in cassava leaves

*In silico* perturbation of PEPC activity demonstrated the metabolic responsive mechanism in leaves that possibly rescued cassava from the stress of water limitation. An increase in PEPC activity led to a rise in the CO_2_ concentration in mesophyll *via* PEPCK and OPPP; the accumulated CO_2_ was then re-assimilated by RuBisCO. The altered carbon flux resulted in metabolic reprogramming in energy metabolism, nitrogen assimilation, sugar metabolism, and biomass biosynthesis. The overall response caused a reduction in leaf growth as more carbon flux was allocated to sink tissues. The simulated mechanism was consolidated by meta-transcriptome data (Ding et al., 2019) that highlighted the mechanistic scenarios in cassava leaves based upon the expression of the related enzymatic genes under drought exposure.

**Figure 9:**
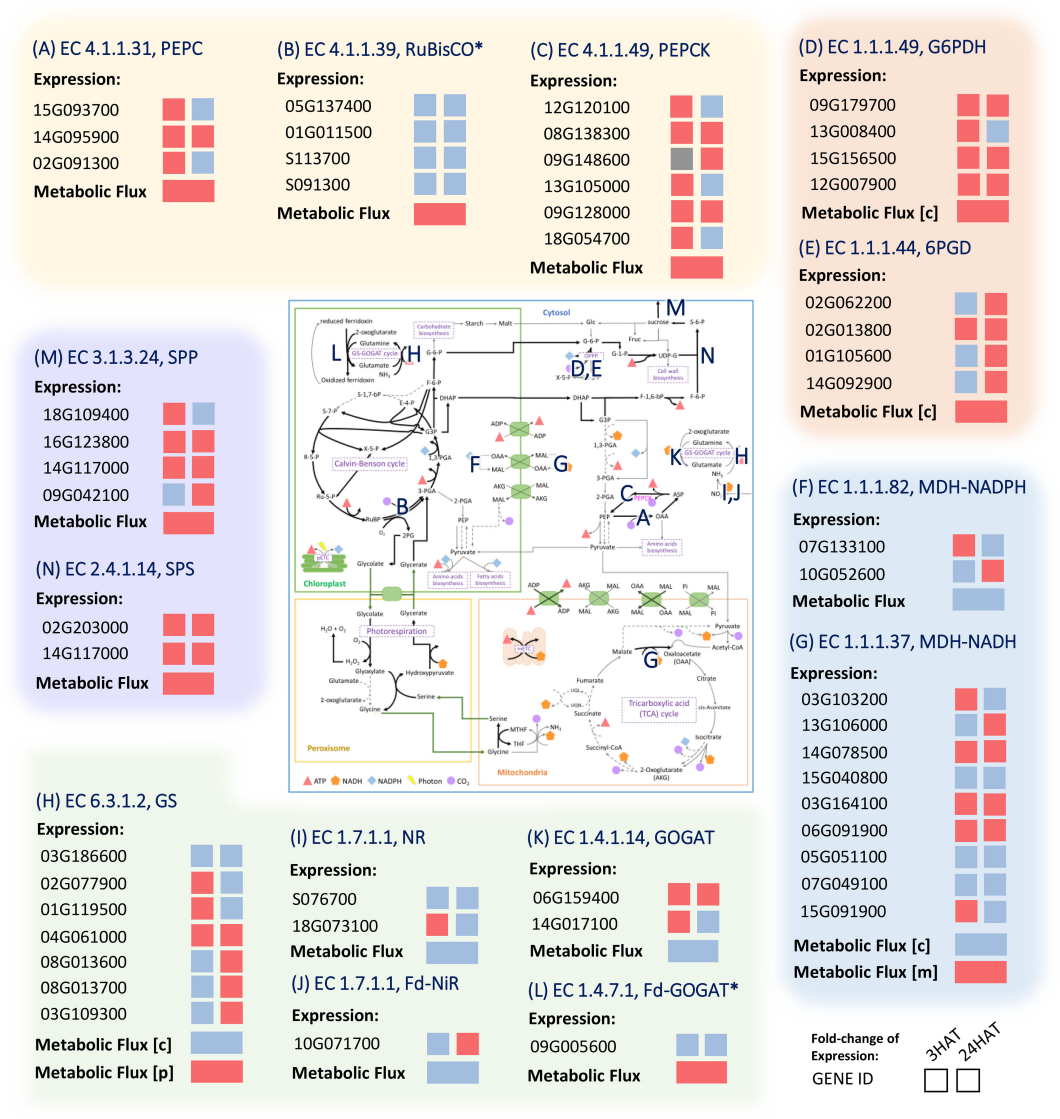
Comparison of the metabolic gene expression in response to drought stress and metabolic activity predicted by the leaf-MeCBM model. Red and Blue represent up- and down-regulated expression or flux in drought, respectively. Grey indicates the undetectable expression in a condition. The E.C. number is associated with a reaction on the map of primary metabolism. **(A)** PEPC; Phospho*enol*pyruvate carboxylase **(B)** RuBisCO; Ribulose 1,5-Bisphosphate Carboxylase-Oxygenase **(C)** PEPCK; Phospho*enol*pyruvate carboxykinase **(D)** G6PDH; Glucose-6-Phosphate Dehydrogenase **(E)** 6PGH; 6-Phosphogluconate Dehydrogenase **(F)** MDH-NADPH; Malate Dehydrogenase (NADP) **(G)** MDH-NADH; Malate Dehydrogenase (NAD) **(H)** GS; Glutamine Synthetase **(I)** NR; Nitrate Reductase **(J)** Fd-NiR; Ferredoxin-Nitrite Reductase **(K)** GOGAT; Glutamate Synthase **(L)** Fd-GOGAT; Ferredoxin-dependent Glutamate Synthase **(M)** SPP; Sucrose Phosphate Phosphatase **(N)** SPS; Sucrose Phosphate Synthase. The expression data were taken from experiments on cassava leaves after 3 and 24 hours of drought treatment (HAT) (Ding et al., 2019). Asterisks denote the infinitesimal changes in gene expression between the conditions, i.e., expression ratio ~ 1. The enzymes are abbreviated in blue and listed in **Supplementary Table 3**.

Under drought conditions, PEPC (Manes.15G093700, Manes.14G095900, and Manes.02G091300) and PEPCK (Manes.08G138300 and Manes.09G128000) genes were increasingly expressed in the last three hours after treatment (HAT), with PEPCK expression still high after 24 hours (**Figures 9A–C**). It indicated a rapid, responsive action of PEPC genes to drought stress and also the associative pathway of action *via* PEPCK. The PEPC reaction metabolized inorganic carbon in the form of HCO_3_^-^ to generate OAA, whereas PEPCK was responsible for converting OAA to PEP and releasing CO_2_. The upregulation of glucose-6-phosphate dehydrogenase (G6PD) (Manes.09G179700, Manes.13G008400, Manes.15G156500, and Manes.12G007900) and 6-phosphogluconate dehydrogenase (6PGD) (Manes. 02G062200, Manes.02G013800, Manes.01G105600, and Manes.14G092900) corresponded to the predicted contribution in providing additional CO_2_ and the associated process of metabolic re-adjustment in the oxidative pentose phosphate pathway (**Figures 9D, E**). The leveled expression of RuBisCO (Manes.05G137400, Manes.01G011500, Manes.S113700, and Manes.S091300; ~ a 0.95-fold change in gene expression) well agreed with the metabolic responses of maintaining CO_2_ abundance and carbon fixation while the stomata remain closed due to stress (**Figure 9B**). The experimental data agree with our simulations of the enzyme’s potential roles in drought response.

Metabolic reprogramming of energy-related metabolism was highlighted by expression profiles of genes belonging to cellular respiration. The upregulation of OPPP genes pointed out the contribution of drought stress to energy homeostasis since OPPP mainly generates cytosolic NADPH (**Figures 9D, E**). The attenuation of the energy allocated from chloroplast to cytosol was in line with the lower expression of the NADPH-dependent malate dehydrogenase (MDH-NADPH (Manes.07G133100 and Manes.10G052600); **Figure 9F**), which is typically located in the chloroplast. In addition, the lower generation of NADH through NADH-dependent malate dehydrogenase (MDH-NADH) in cytosol agreed with the lower expression levels of some genes belonging to the MDH-NADH enzyme (**Figure 9G**). In mitochondria, the expression levels of genes responsible for the mitochondrial electron transport chain (mETC, data not shown) and other genes belonging to the MDH-NADH enzyme were increased during the response to drought. That was consistent with the high flux of malate entering the mitochondria for NADH production through the mitochondrial NADH-MDH. The produced NADH was subsequently used for producing ATP through the mETC, as indicated by its up-regulated expression in drought conditions. Together, the concordance between the experimental data and the predicted metabolic patterns revealed cellular adjustments in energy and redox homeostasis during drought stress response.

The gene transcription profiles also suggested metabolic reprogramming of carbon and nitrogen metabolism in response to drought stress. The increased expression of sucrose phosphate phosphatase (SPP; Manes.18G109400, Manes.16G123800, Manes.14G117000, Manes.09G042100, Manes.08G037000, and Manes.10G071200); **Figure 9M**) and sucrose phosphate synthase (SPS; Manes.15G055400, Manes.02G203000, and Manes.14G117000; **Figure 9N**) supported the predicted greater tendency of carbon partitioning to sucrose metabolism under drought conditions. In addition, lower expression of most genes related to biomass pathways (data not shown) was consistent with the decreased fluxes throughout biomass synthesis pathways. The lower expression level of most genes related to nitrate reductase (NR; Manes.S076700 and Manes.18G073100; **Figure 9I**), ferredoxin-nitrite reductase (Fd-NiR; Manes.10G071700; **Figure 9J**), and glutamine synthase (GS, **Figure 9H**) under drought conditions corresponded to the lower nitrogen assimilation in the cytosol. Altogether, the expression data corroborate our findings on metabolic adjustment toward energy-redox metabolism, nitrogen metabolism, and sucrose biosynthesis during the response to drought stress.

## 4 Discussion

**Figure 10:**
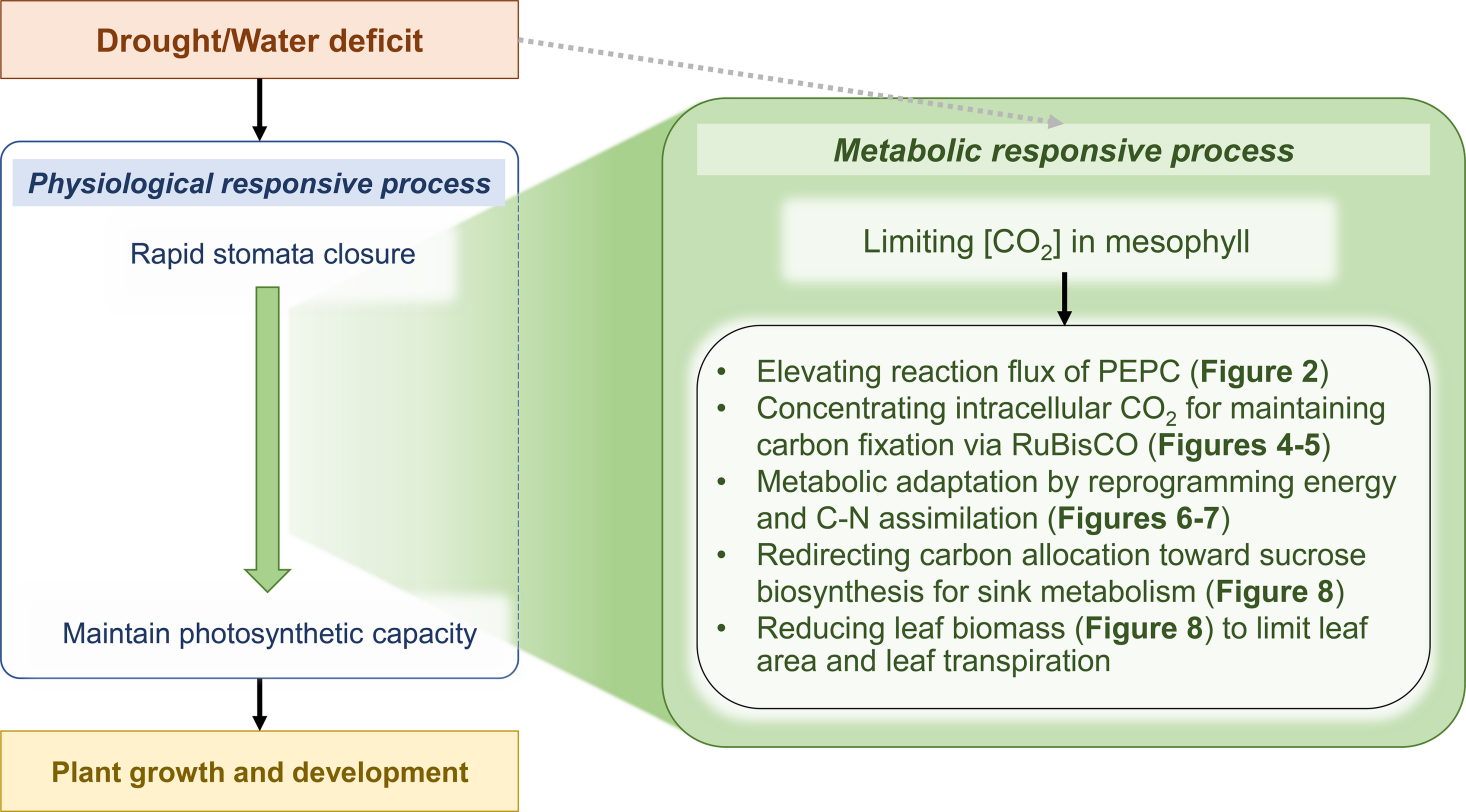
Scheme of physiological and metabolic responses acting in concert against drought stress in cassava leaves. Green represents the proposed metabolic response mechanisms acting in concert with the physiological response to enable tolerance and maintain normal cassava growth and development in a water-deficit environment. Grey dashed arrow indicates that the model did not consider a direct effect of drought on entire enzyme activities.

Drought is a major stress factor that harms plant growth and yield, even in highly tolerant crops such as cassava. PEPC is a primary step of CO_2_ assimilation in the photosynthesis of C_4_ and CAM (Crassulacean Acid Metabolism) plants. It catalyzes PEP and HCO3- to form the C_4_ metabolite oxaloacetate (OAA), which is later decarboxylated to release CO_2_ for RuBisCO. The mechanism was proposed to enhance the acclimation of plants to relatively hot and dry environmental conditions (Leegood, 2002). During a sharp decline of intercellular CO_2_ concentration due to stomata closure, the CO_2_-concentrating mechanism suppresses photorespiration (oxygenation) during CO_2_ fixation by RuBisCO. In photosynthetic tissues of typical C_3_ plants, the fundamental function of PEPC is to replenish tricarboxylic acid cycle intermediates that are withdrawn for a variety of biosynthetic pathways and nitrogen assimilation (Chollet et al., 1996; Izui et al., 2004). The production of malate, a C_4_ organic acid, is thought to be directly linked to carbon metabolism, as PEP, the substrate for carboxylation, originates mainly from carbon skeletons. In guard cells, the higher PEPC activity participates in stomatal movement through malate accumulation, which contributes to charge balance (as anion) and maintains membrane potential during stomatal opening (Vavasseur and Raghavendra, 2005; Cousins et al., 2007). In this work on computational modeling of cassava leaves, leaf-MeCBM was constructed and employed to study metabolic responses to drought in concert with physiological responses. The model sheds light on the role of PEPC enzyme in facilitating metabolic tolerance to drought. Simulations showed that the lower CO_2_ uptake rate, representing stomatal closure under dry conditions, induced flux through PEPC, supporting CO_2_ fixation by RuBisCO. This metabolic response was further demonstrated through the computational perturbation experiment of PEPC activity. The *in silico* elevated PEPC activity increased the intracellular CO_2_ pool, which then was assimilated by RuBisCO. It affected the downstream metabolism by changing carbon flux routes and energy homeostasis. It finally lowered the biomass production and increased the sucrose biosynthesis. The simulation showed highly consistent with the transcriptome data of cassava leaves under a water-stress condition, which certified the hypothetical role of PEPC proposed herein. The findings allowed us to explore the systematic synchronization of plant physiological and metabolic responses to drought stress (**Figure 10**).

Cassava has relatively high PEPC expression comparing to a typical C_3_ plant, especially under hot and dry climates (Lopez et al., 1993; El-Sharkawy et al., 2008). Without the Krantz anatomy, PEPC was proposed to play a role in recycling and concentrating CO_2_ for RuBisCO when stomata closed from stress. The most recent physiological data showed that photosynthesis and carbon fixation of cassava behaves more closed to C_3_ plants, yet having exceptional advantageous mechanism from C_4_ plants (Edwards et al., 1990; Angelov et al., 1993; Arrivault et al., 2019). The leaf-MeCBM, therefore, represented the metabolic process in cassava leaves by assuming mainly a C_3_-plant metabolism. The atmospheric CO_2_ was first fixed by RuBisCO (via Calvin-Benson cycle), and then produced a three-carbon acid (i.e., 3-phosphoglycerate, 3PGA). The primary metabolism of cassava leaves was assumed to take place primarily in mesophyll cells across four sub-cellular compartments: cytosol, chloroplast, mitochondria, and peroxisome. The C_4_-photosynthesis pathway in cassava was modelled according to the presence of the related genes in cassava genome and the cassava literature. It was composed of PEPC in cytosol, NADP-ME in the chloroplast, NAD-ME in the mitochondria, and PEPCK in the cytosol. The leaf-MeCBM showed that PEPC pathway was upregulating and involved in maintaining the steady state of photosynthetic carbon fixation under simulated drought condition (**Figure 3**) (El-Sharkawy and Cock, 1987a). The simulation corresponds to the persistent photosynthesis capability in cassava plants grown under the free-air CO_2_ enrichment (FACE) condition, though stomatal conductance was observed reducing. These plants showed lower specific leaf area, but higher final harvest root yield with respect to the atmospheric CO_2_ condition (Forbes et al., 2020; Ruiz-Vera et al., 2021). Similarly, PEPC-overexpression in rice showed higher net photosynthetic rates with unchanging yield under drought condition compared to wild-type (Zhou et al., 2011; Zhang et al., 2017).

*In silico* elevating PEPC activity helped elucidate the systematic response of cassava leaf metabolism as a consequence of the metabolic reprograming potentially happened during stomatal closure. The increase in PEPC activity demonstrated the alteration in carbon fluxes throughout the whole metabolism (**Figure 3**). Modulation of nitrogen and sugar metabolism along with PEPC activities in the model was implied by the associated adjustments of C and N assimilation to meet the needs of metabolism (Shi et al., 2015) (**Figure 7**). The higher fluxes through the GS/Fd-GOGAT cycle (nitrogen metabolism) and serine metabolism in chloroplast (**Figure 7B**) indicated a coordination of PEPC activity and N metabolism through an alteration in photorespiration pathway. The predicted highly active sucrose biosynthesis pathway (**Figure 8B**) infers the metabolic tendency to increase osmolyte production. Accumulation of these molecules, including soluble sugars, is reported to rescue plants from drought through adjusting the ROS balance, and restoring the osmotic balance by reducing leaf transpiration under stress exposure (Singh et al., 2015). However, the alteration in carbon fluxes towards the sucrose-related pathway resulted in decreased biomass synthesis rate (**Figures 3** and **8A**). The greater partitioning of carbon to sucrose biosynthesis than biomass production reflected the response of leaf (source) metabolism to secure more resource in the storage organs. This trade-off between sucrose accumulation and growth under stress has been reported in ranging crop plant species (Lawlor and Cornic, 2002; Chaves et al., 2009), and also helps explain the metabolic phenomena underlying a rapid drop of cassava leaves, once exposed to stress. The *in-silico* high sucrose systhesis may promote photoassimilate translocation to storage organs (Chaves, 1991; Erin and Brent, 2012), resulting in a high final-harvest index under prolonged drought (El-Sharkawy, 2007).

In rice (*Oryza sativa*), the chloroplast-located PEPC isoform was reported to play a crucial role in nitrogen metabolism as found the alteration in ammonium assimilation and amino acid synthesis (Masumoto et al., 2010). The transgenic rice plants over-expressing maize C_4_ PEPC gene also exhibited a higher soluble sugar content (sucrose, glucose, and fructose) in both control and drought conditions. PEPC was proposed to alleviate oxidative damage and be more related to high endogenous saccharide decomposition that enhanced drought tolerance in rice plants (Zhang et al., 2017). Corresponding to the study in *Arabidopsis*, the suppression of PEPC genes showed a decrease in amino acid biosynthesis, especially GS/GOGAT cycle and NH_4_^+^ assimilation. The alteration in starch and sucrose accumulation also found in the *Arabidopsis* transgenic lines, indicating the effect of PEPC on C/N balance in leaf metabolism (Shi et al., 2015).

In addition to the metabolic insights, this work also highlighted the importance of computational modeling in facilitating the study of complex systems. The model demonstrated the plausibility of the hypothetical action of PEPC in cassava metabolism and helped clarify the conceptual roles of PEPC proposed in cassava literature. Modelling conversion of carbon metabolites in cassava leaves using leaf-MeCBM could help connect the physiological and metabolic responses of cassava to the prevailing drought environment. Nonetheless, all simulations and interpretation from leaf-MeCBM was based on the assumption of C_3_-metabolism in cassava leaves. While the simulated results and findings were promising and well correlated with the transcriptome data and cassava literature, further experiments on intracellular response are required to narrow down the gap of conceptual insights to the real occurrence in plant cells.

## 5 Conclusions

In summary, the *in silico* metabolic modeling using leaf-MeCBM revealed that the enzyme PEPC plays a crucial role in concentrating the internal CO_2_ and enhancing CO_2_ fixation by RuBisCO. Carbon (e.g., sucrose) and nitrogen metabolism and balance proved critical for drought stress tolerance. This work also captured the metabolic mechanism behind energy and redox homeostasis. The overall metabolic reprogramming resulted in high sucrose production but a low biomass production rate. This work provides a holistic view of the leaf metabolic and physiological responses of cassava to drought stress. The insights gained could be leveraged to develop tolerant cultivars. Ultimately, this model could also be extended to explore the source-sink relationship and guide metabolic engineering toward enhancing yield and abiotic stress tolerance in cassava and other crops.

## Data availability statement

The original contributions presented in the study are included in the article/**Supplementary Materials**. Further inquiries can be directed to the corresponding author.

## Author contributions

TS conceived and designed the experiment. TS and SK supervised the project. NP developed the model structure, performed all computational analysis, and prepared the figures. TS and NP analyzed and discussed the results. SK discussed the results. NP and TS drafted the manuscript. All authors contributed to the article and approved the submitted version.

## Supplementary material

Supplementary Data Sheet 1 The reconstructed genome-scale metabolic model of cassava leaf (leaf-MeCBM).

Supplementary Figure 1 Scheme of the simplified carbon assimilation metabolic pathway in the leaf-MeCBM model.

Supplementary Figure 2 The simulated leaf growth rate and specific CO2 uptake rate.

Supplementary Figure 3 Simulation of PEPC reaction flux during reduced CO2 uptake and increased level of photorespiration.

Supplementary Table 1 Characteristics of the leaf-MeCBM model.

Supplementary Table 2 Leaf-MeCBM simulation of characteristic behaviors of leaf metabolism.

Supplementary Table 3 Abbreviation and full names.
