# Supplementary material for: CO2 recycling by phosphoenolpyruvate carboxylase enables cassava leaf metabolism to tolerate low water availability
Source: Front Plant Sci. 2023 May 9;14:1159247. doi: 10.3389/fpls.2023.1159247 (PMC10204807; doi:10.3389/fpls.2023.1159247)
Supplement: Supplementary Figure 2 — The simulated leaf growth rate and specific CO2 uptake rate. [file Image_2.pdf]

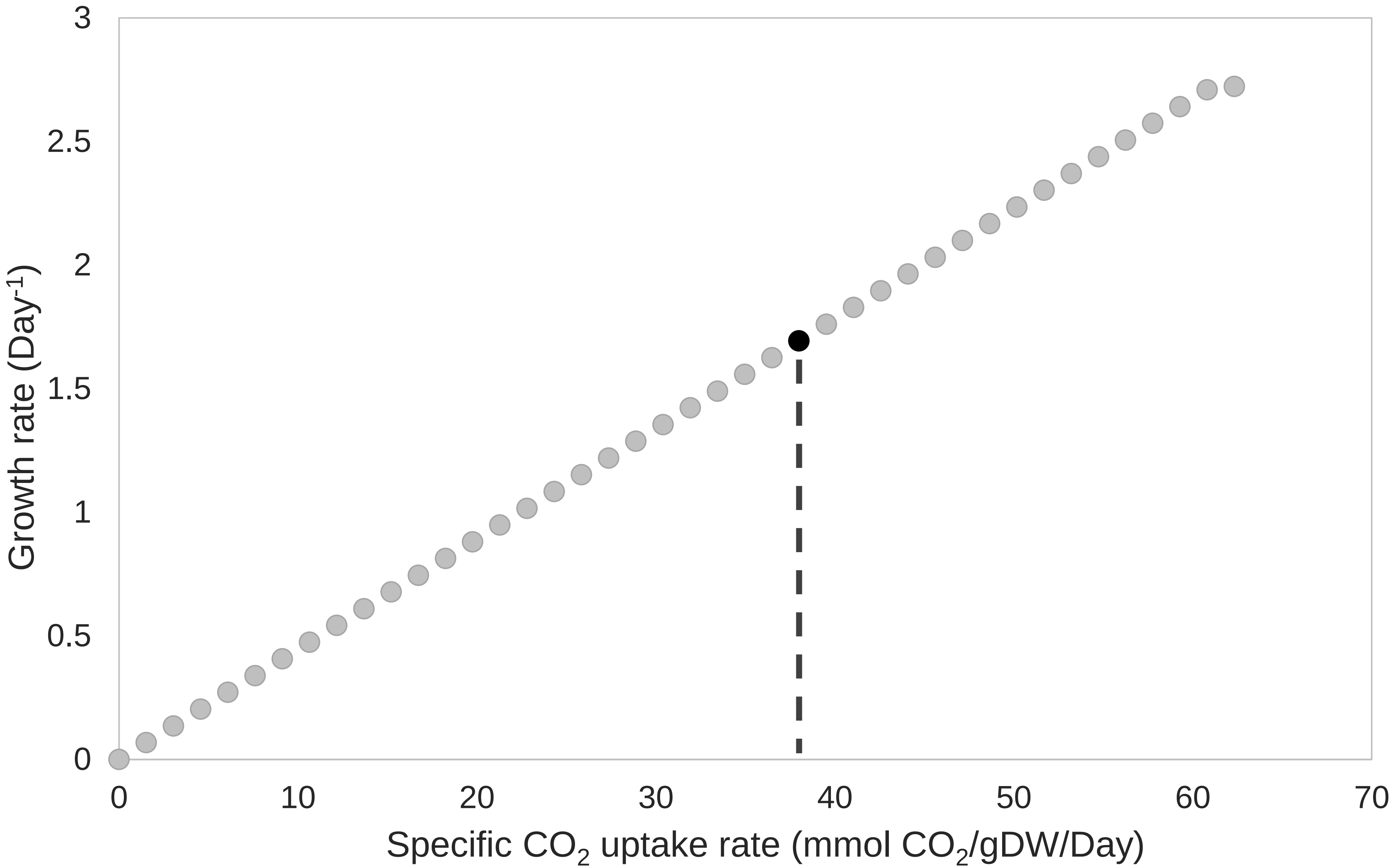

Supplementary Figure S2 The simulated leaf growth rate and specific CO_2 uptake rate. The leaf-MeCBM model simulation under the studied condition is marked in black.
