# Supplementary material for: CO2 recycling by phosphoenolpyruvate carboxylase enables cassava leaf metabolism to tolerate low water availability
Source: Front Plant Sci. 2023 May 9;14:1159247. doi: 10.3389/fpls.2023.1159247 (PMC10204807; doi:10.3389/fpls.2023.1159247)
Supplement: Supplementary Figure 3 — Simulation of PEPC reaction flux during reduced CO2 uptake and increased level of photorespiration. [file Image_3.pdf]

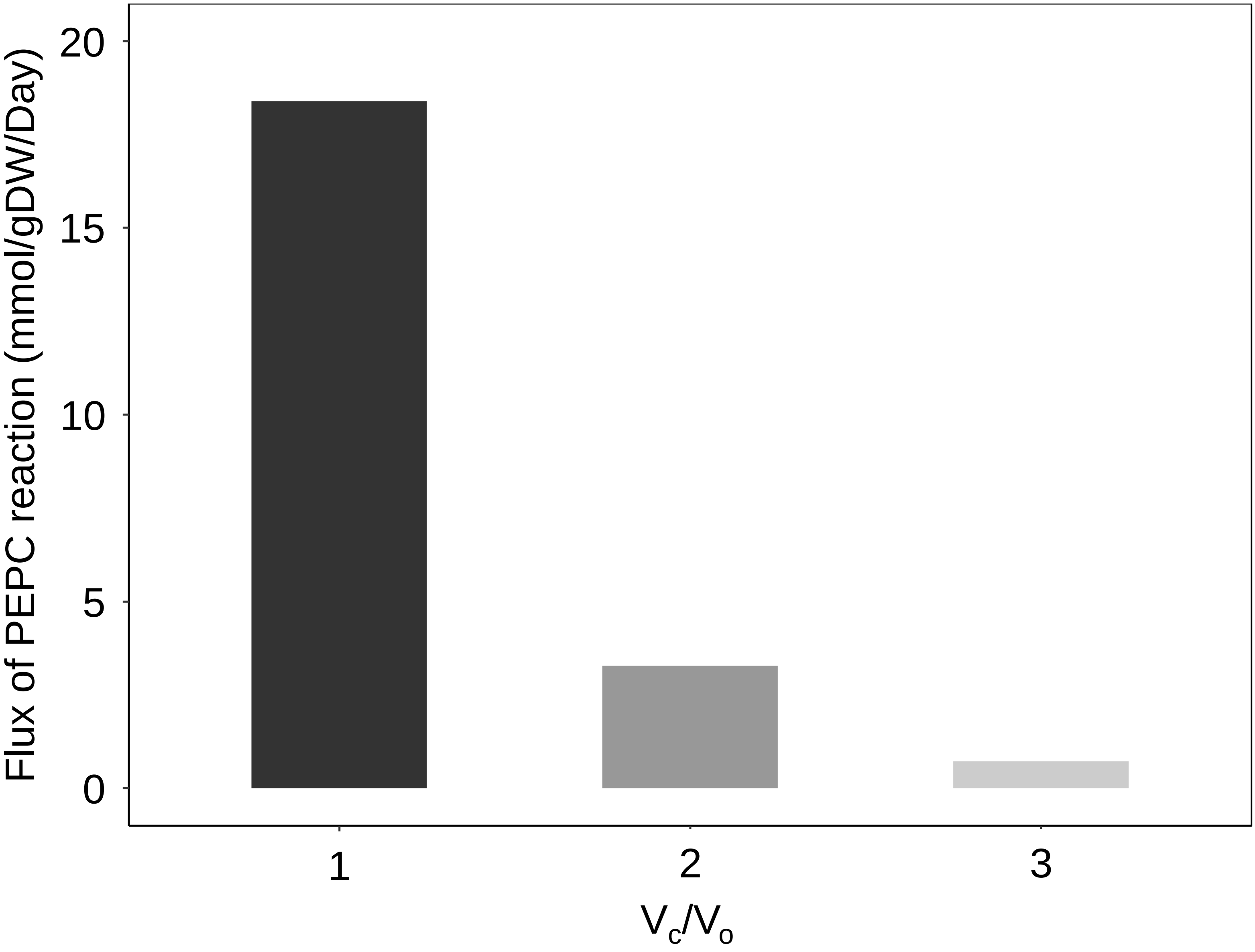

Supplementary Figure S3 Simulation of PEPC reaction flux during reduced CO_2 uptake (50% of normal condition) and increased level of photorespiration (V_c/V_o ratio < 3).
