# Supplementary material for: CO2 recycling by phosphoenolpyruvate carboxylase enables cassava leaf metabolism to tolerate low water availability
Source: Front Plant Sci. 2023 May 9;14:1159247. doi: 10.3389/fpls.2023.1159247 (PMC10204807; doi:10.3389/fpls.2023.1159247)
Supplement: Supplementary Figure 1 — Scheme of the simplified carbon assimilation metabolic pathway in the leaf-MeCBM model. [file Image_1.pdf]

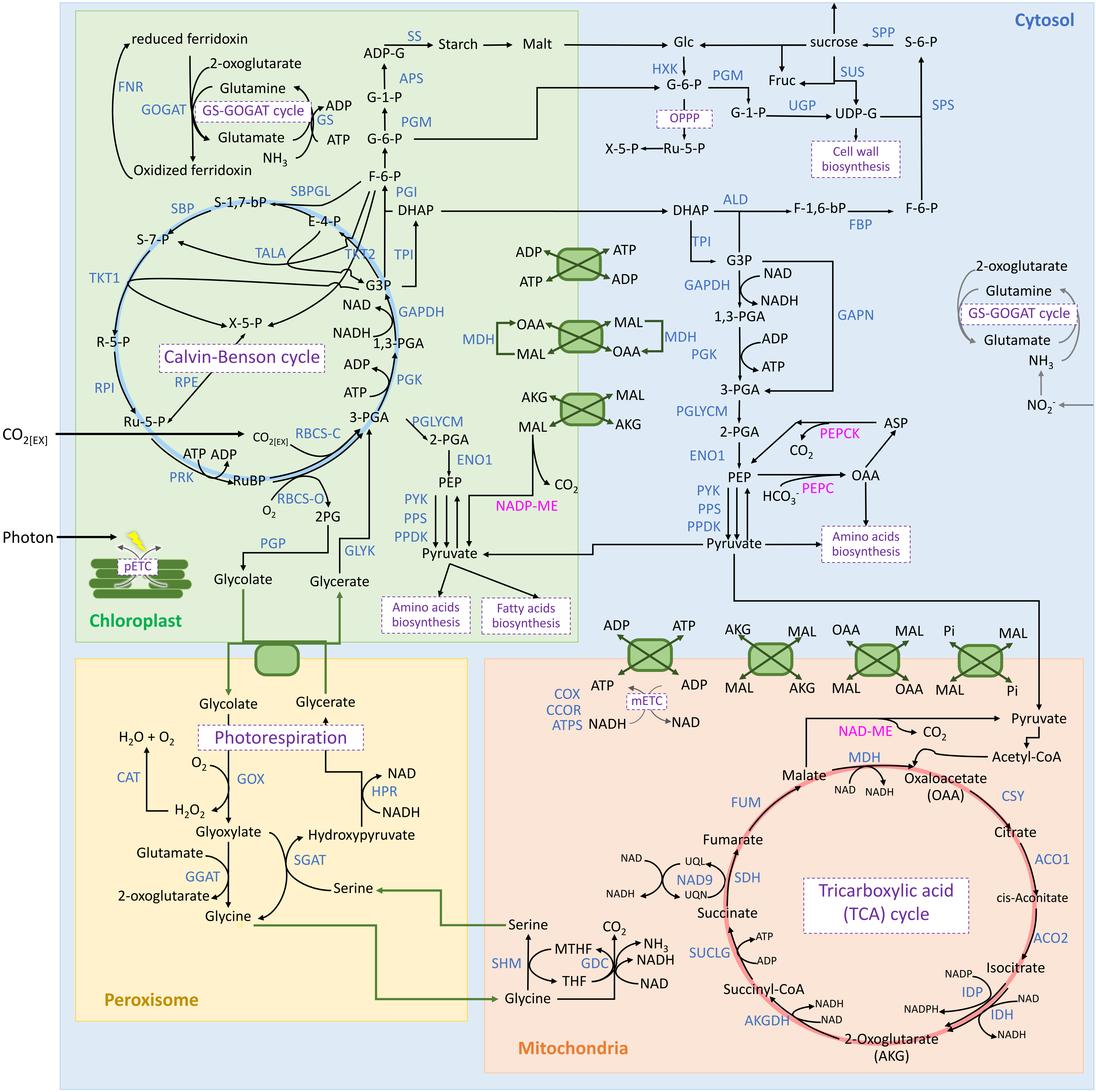

Supplementary Figure S1. Scheme of the simplified carbon assimilation metabolic pathway in the leaf-MeCBM model. The enzymes are abbreviated in blue and are listed in Supplementary Table S3. Three subtypes of C_4 photosynthesis are highlighted in pink. Green ovals indicate subcellular transporters. Metabolic pathways are denoted in purple. The background colors depict subcellular compartments, blue – cytosol, green – chloroplast, orange – mitochondria, and yellow – peroxisome.
